# Low Energy Physical Activity Recognition System on Smartphones

**DOI:** 10.3390/s150305163

**Published:** 2015-03-03

**Authors:** Luis Miguel Soria Morillo, Luis Gonzalez-Abril, Juan Antonio Ortega Ramirez, Miguel Angel Alvarez de la Concepcion

**Affiliations:** 1 Computer Languages and Systems Department, University of Seville, 41012 Seville, Spain; E-Mails: jortega@us.es (J.A.O.R.); maalvarez@us.es (M.A.A.C.); 2 Applied Economics I Department, University of Seville, 41018 Seville, Spain; E-Mail: luisgon@us.es

**Keywords:** contextual information, mobile environment, discretization method, qualitative systems, smart-energy computing

## Abstract

An innovative approach to physical activity recognition based on the use of discrete variables obtained from accelerometer sensors is presented. The system first performs a discretization process for each variable, which allows efficient recognition of activities performed by users using as little energy as possible. To this end, an innovative discretization and classification technique is presented based on the *χ*^2^ distribution. Furthermore, the entire recognition process is executed on the smartphone, which determines not only the activity performed, but also the frequency at which it is carried out. These techniques and the new classification system presented reduce energy consumption caused by the activity monitoring system. The energy saved increases smartphone usage time to more than 27 h without recharging while maintaining accuracy.

## 1. Introduction

**Figure 1.**
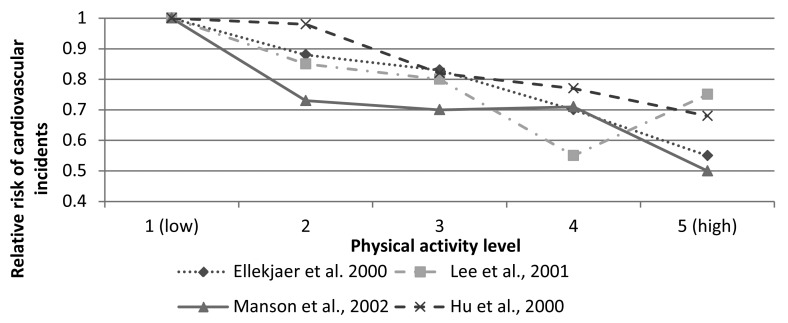
Associations between the risk of cardiovascular incidents and physical activity level.

Just 30 min of moderate activity five days a week can improve your health, according to the Centers for Disease Control and Prevention. By enabling activity monitoring on an individual scale over an extended period of time in a ubiquitous way, physical and psychological health and fitness can be improved. Studies performed by certain health institutes [1–4] have shown significant associations between physical activity and reduced risk of incident coronary heart disease and coronary events. Their results can be seen in Figure 1, where the inverse correlation between the risk of cardiovascular incidents and physical activity level is shown through a comparison of four separate studies.

In recent years, thanks largely to increased interest in monitoring certain sectors of the population, such as elderly people with dementia and people in rehabilitation, activity recognition systems have increased in both number and quality Furthermore, communication between relatives, friends and professionals can be improved by means of graphs of weekly activity (highly relevant for sportsmen and relatives of elderly people), whereby the doctor can be automatically alerted if any strange activity is detected. In fact, automatic recognition of human activity represents one of the most important research areas in ubiquitous computing [5,6]. For this reason, it is extremely important to ensure that the intrusion level caused by the system is the lowest possible. Some recent works, such as [7–9], attempt to solve this problem by using a variety of sensors, such as accelerometers, gyroscopes, GPS and even radio-frequency identification and near-field communication (NFC) sensors.

As will be seen below, however, the use of these sensors causes a major drain on the energy of autonomous devices running on batteries, such as smartphones. On the other hand, by using data acquired from these sensors and applying certain classification methods, it is possible to perform pervasive physical activity monitoring. Some of these algorithms, such as Bayesian decision (BDM), decision tree algorithms (RBA), least-squares methods (LSM), support vector machines (SVM), K-nearest neighborhood (KNN) and artificial neural networks (ANN), will be analyzed and compared in this work. The results show the main differences between different studies, and certain drawbacks will be determined. These drawbacks, commonly related to energy consumption and computational cost, do not make possible their implementation on real users' smartphones. The first difference observed between the systems developed so far is the type of sensor used.

There are systems that use specific hardware [10–12], whereas others use general purpose hardware [7,13,14]. Obviously, the use of generic hardware, as smartphones, is a benefit to users, because the cost of such devices and their versatility are assets in their favor. The risk of loss, forgetting and disuse is decreased because users' smartphones have already been integrated into the users' daily life. However, general purpose devices are used for other purposes, such as making phone calls, surfing the Internet and listening to music. For this reason, the physical activity recognition system must be executed in background mode and should cause the least impact as possible on the system in terms of complexity and energy consumption.

Another difference found among related studies is the number and position of sensors used. In [15], the accelerometer sensor is placed in a glove, which the user must wear. This sensor can recognize a multitude of activities, depending on the movement of the hand. In contrast, some studies use diverse sensors all over the body to recognize these activities [16–18]. In recent years, as a result of technological progress, it has been possible to build sensors of reduced size that can be installed into the user's clothes [19] or attached to the user's body [20,21]. According to some comparative studies and research based on multiple sensors, this type of sensor gives higher accuracy, although [13] shows that it is more comfortable for the user when the sensor is placed into the user's pocket or at the hip. This increased user acceptance for devices attached at the hip or in the pocket is because the installation on a monitored person's body is easier, not to mention that the infrastructure is much more simple and inexpensive. Once the most comfortable alternative for users is determined, some device sensors can be chosen to perform the activity monitoring.

Some works, close to social computing, make use of microphones [22–26] and electrocardiogram (ECG) sensors [27–29] for this purpose. The former type, which consists of microphone and Bluetooth devices, helps to obtain contextual information about the user's environments and would be appropriate to perform a deeper analysis of the activity, for instance if the user is walking in a disco or at home, if the user is alone or with someone. However, high-level activity recognition (walking, playing, running or standing up) is done using other sensors. ECG can help in determining high-level activities by means of heart rate processing. In this sense, some activities (walking or running) could be discerned based on the effort needed to perform them. The problem here is that ECG sensors are expensive and uncomfortable for the user.

In other works [21], data for activity recognition are obtained through any kind of mobile device (not only mobile phones), although these data are sent to a server, where the information is subsequently processed. Thus, the computational cost is not a handicap, as learning and/or recognition are performed in the server and a more complex processing can be applied. In contrast, when processing is carried out in the mobile device itself [30], efficiency becomes a crucial issue. In this vein, in order to apply a solution based on distributed computing, the device must always be connected to a data network. This does not currently represent a major drawback, since most devices have this kind of connectivity, although there are still users (mostly elderly) whose devices have not been associated with a continuous data connection outside the range of WiFi networks. Finally, decrease the energy cost conflicts with the need to send the data collected in a continuous way between device and server. This means that current strategies of sensor batching (as will be seen hereafter) cannot be applied, and devices must be continually waking up from sleep mode. Furthermore, the intensive use of the data network has a deep impact on the energy use. The work in [31] shows the increase in energy consumption when 3G and WiFi are used, and in [32], it can also be observed that approximately 44% of battery usage in smartphones occurs by the use of GSM (3G or 2G).

Taking into account previous works, physical activity monitoring through smartphones presents the following challenges:

- To decrease as far as possible the risk of forgetting the processing device, so as to carry out continuous monitoring of users, everywhere and anytime;
- To reduce the energy impact on the smartphone, developing an accurate and efficient system;
- To integrate learning and monitoring on the device itself, in real time and without server information sharing.

Along this work, all of these challenges are addressed, and certain solutions are offered to achieve the proposed objective: to build a complete, accurate and low energy consumption system for pervasive physical activity monitoring using sensors embedded on smartphones.

The remainder of this paper is organized as follows. Section 2 presents the need to reduce the energy consumed by physical activity monitoring systems when they are executed in a smartphone. Section 3 presents the feature extraction model, the dataset obtained from sensors and all physical activities that can be recognized by the described system. In Section 4, discretizing continuous variables and a classification process are presented. Section 5 compares the presented method and other methods used previously in the literature. Finally, Section 6 discusses the advantages of the proposed algorithm, as well as some challenges and future works about the recognition system described.

## 2. Justification for the Reduction of Energy Consumption

Applying Moore's law, manufacturers increase processing power at least twice each year, in contrast with battery development, which did not even double over the last five years. This is not secondary at all. From a survey performed by North American Technologies [33], battery life is the second most important purchase decision factor for smartphones. Users' acceptance of context-aware applications in general and of activity monitoring systems in particular is critical. For this reason, not only has an accurate and fast system been developed, but a low energy consumption model from the viewpoint of discrete techniques is also presented throughout this work.

To determine the building blocks that promote the least drain of energy, a comparison between the energy consumption of the most frequently-used smartphone sensors in the literature is made. This is critical for choosing the most efficient sensors to be used in the monitoring application. Although some of these sensors cannot be used separately to determine the physical activity performed, some of them could work together.

An application has been developed to measure the battery energy consumed by each sensor in a real environment. For this purpose, Samsung Galaxy S2, Nexus One, Samsung Galaxy S3, Nexus 5 and HTC Tatoo were used. The application was run for four weeks, with battery consumption calculated based on the activated sensors. To prevent problems arising from the use of concrete devices, the application was installed on 20 users' smartphones with different features. The eight most-used sensors (microphone, GPS, WiFi, accelerometer, NFC, Bluetooth, ECG connected by Bluetooth and gyroscope) from the literature in the field of activity recognition were analyzed. It must be taken into account that battery consumption for the microphone depends not only on the microphone sensor itself, but also on the sample rate and the buffer. The last one is useful to keep the audio codec, memory controller and DMA engines awake for the shortest possible time.

**Figure 2.**
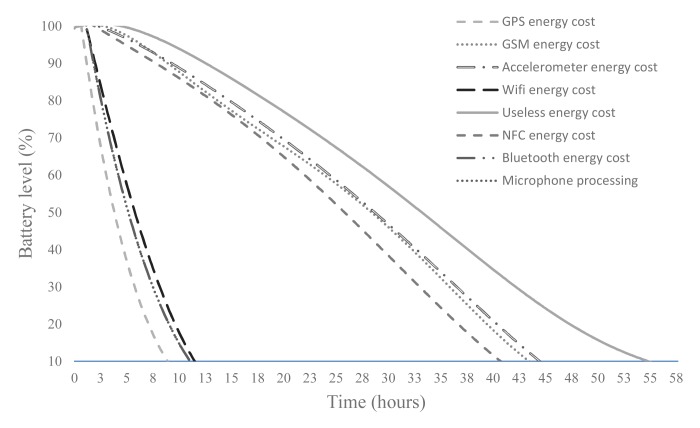
Energy cost comparison between sensors embedded in smartphones.

To avoid battery capacity and the energy expenditure of the smartphone without performing any action and with no user interaction, a useless energy cost trend line is generated. This line (generated in the absence of normal user usage) is useful as the baseline, regardless of the time and the power consumption of sensors over several kinds of smartphones with different features. Figure 2 shows the result of this comparison (The values are displayed in hours regarding a generic device. However, during the evaluation process, the lifetime deviation compared to the values of useless energy cost has been studied on different devices), with the time represented on the horizontal axis and the battery level at a specific instant of time appearing on the vertical axis. The procedure to represent the results was as follows. The useless energy cost is measured for all devices on which the study was conducted. Once this value is measured (associated with 100% of the battery lifetime), the rest of the battery time runs using different sensors (GPS energy cost, accelerometer energy cost, and so on) was obtained. By a simple ratio, the percentage of reduction in the lifetime of the battery relative to the baseline (useless energy cost) is calculated. Thus, an approximate percentage of the impact that sensors have on the battery lifetime was obtained. Finally, to illustrate the results, these percentages are reflected on a generic device where the unused battery time is about 56 h. It can be seen in the figure that the lowest power consumption is given by the microphone, followed by the accelerometer sensor. Therefore, from these results, it can be deduced that the use of GPS or Bluetooth does not constitute a good choice to develop an energy-efficient physical activity monitoring system, despite their having higher accuracy. In the case of Bluetooth, advances in this sensors have reduced the energy consumption, but this technology still suffers from serious problems when being used in the field of activities recognition. On the one hand, the infrastructure must be installed in each location where it will be used. Currently, there are just a few public Bluetooth access points. Furthermore, dynamic activities, such as walking, running or cycling, can hardly be recognized by Bluetooth, unless additional devices associated with these activities are installed on the objects (bike, skateboard, and so on). It must be noted that nowadays, the smartphone is the only device (together with certain wearables, such as smart-watches) carried continuously for most users. Therefore, the use of Bluetooth devices for activity recognition systems must force the use of these devices, which would not be suitable for user acceptance of AR (*Activity Recognition*) systems. Finally, the cost of infrastructure is also a determining factor. Bluetooth access point networks are more expensive than embedding all of the necessary technology in the smartphone itself.

Other research is based on the use of microphone and voice recognition to determine the context of the user [34], and it could be thought that the result is more energy efficient. However, voice recognition presents problems when the environment is noisy or the user is alone, so sometimes, it is not possible to obtain results from the audio signal classifier. Thus, in the cited work, this process was complemented with other methods based on inertial measurement units (IMUs). It must be noted that an IMU is a device that measures velocity, orientation and gravitational forces, using a combination of accelerometers, gyroscopes and magnetometers.

To reduce the cost related to process accelerometer signals, this paper opts for an innovative technique, through which, the work is performed in the field of discrete variables. Thanks to a discretization process, the classification cost is much lower than that obtained when working with continuous variables. Any dependence between variables during the recognition process is therefore eliminated, and energy consumption from the process itself is reduced.

## 3. Building the Dataset

### 3.1. Embedded Sensor Limitations

Throughout this work, a triaxial accelerometer, BMA150 Bosh, integrated into a Google Nexus S, and other similar accelerometers integrated into a Samsung Galaxy S3 and a Nexus 5 were used. These sensors have a range of sampling frequencies between 25 Hz and 1500 Hz; however, human activities have a relatively low frequency, so it is not necessary to fully exploit the capabilities of the sensor. Activities, such as walking, running and jumping, can be determined with small blocks of data. This choice is also supported by other related works using similar devices [35]. However, when working with these elements, not only the high energy consumption required by the data collection, but also the processing of such data must be taken into account.

Mobile devices currently feature a multitude of sensors that are used routinely. This means that, as a result of high energy consumption, the useful time between device recharges is very low. Thus, usage is conditioned by dependence on the electric grid to recharge the mobile device. On the other hand, there are various solutions using specific hardware [36] that have a high degree of autonomy. The problems faced by these elements, however, include the aforementioned risk of loss and the risk of leaving the hardware behind, together with the discomfort for users. Furthermore, these solutions tend to be very expensive and are not oriented towards a wide range of applications.

### 3.2. Feature Extraction

**Figure 3.**
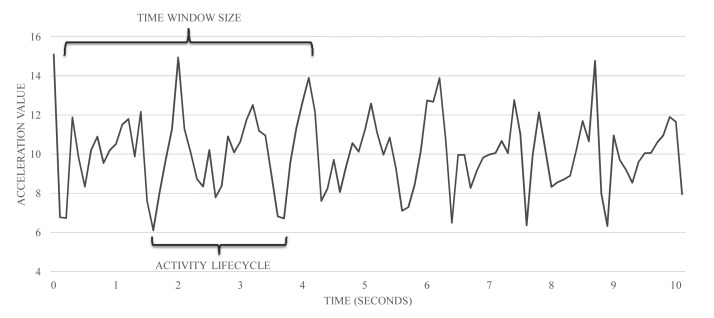
Relation between windows length and activity cycle.

Certain related studies attain results on activity recognition off-line. A comprehensive training set from the inertial sensor output is first needed before data can be classified into any of the recognized activities. For this purpose, both training and recognition sets are obtained using overlapped time windows of fixed duration. Following the conducting of a performance and system accuracy analysis, it is determined that the optimum length for these windows is 4 s with 1 s overlapping [37,38]. The recognition delay is determined by the following relation:

(1) delay=size(υ)⋅0.75+k⋅f(size(υ))

where *size*(*υ*) is the length of the time window, *k* is a computational constant that depends on the specific device and the *f* function represents the discretization and classification complexity, which depends on the length of the time window. The length of each time window has been chosen, because it is very important to ensure that each time window contains at least one activity cycle. Figure 3 shows the segmentation process and activity cycle.

As a premise for the use of time windows as a data collection method, it is assumed that the physical activities recognized by the system must have a duration greater than or equal to the size of this time window. Therefore, certain activities, such as a fall, may not be recognized by the system due to their exceptionality. Once data have been obtained, it is necessary to perform a filtering process before making the classification in order to remove all signal noise. In most cases, the noise of IMUs is negligible, although one kind of noise that can seriously affect the activity classification exists. This noise is produced by vibrations that take place on the device when it is carried by the user. A Butterworth low pass filter is applied to reduce the noise generated. Finally, following this strategy, a total of 561 temporal and frequential variables were derived from the inertial sensors on the devices.

### 3.3. Set of Activities

To improve and establish a comparative baseline for classification algorithms, which is easy and publicly replicable, it was decided to use two public datasets and one built for this study. The first dataset under study, named the Human Activity Recognition Using Smartphones Data Set, presented in UCI [39], is composed of 10,299 instances of time windows sampled in fixed-width sliding windows of 2.56 s and 50% overlap (128 readings/window) at a constant rate of 50 Hz, 561 variables and six activities labeled, carried out with a group of 30 volunteers with an age bracket of 19–48 years. The second public dataset, named the PAMAP2 Physical Activity Monitoring Data Set and published in UCI, as well [40], consists of 2,211,669 RAW data from three IMUs and 18 activities, performed with nine subjects between 26 and 31 years. RAW data were grouped into 8847 overlapped time windows, and system variables were obtained from them. The structure and content resulting from these datasets are very similar. Both are composed of a set of rows, and each row contains the variable values separated by a comma. Some features, such as the mean, standard deviation, median deviation, maximum, minimum, energy, interquartile range, signal entropy, correlation coefficient, skewness, kurtosis, angle between vectors and the Jerkmean, are processed in order to generate the complete dataset from RAW values of the accelerometer and gyroscope (this being understood as data obtained directly from IMUs embedded in the smartphone, *i.e.*, triaxial acceleration from the accelerometer and triaxial angular velocity from the gyroscope).

The users involved in the experiment followed a protocol in which the activities were performed wearing a waist-mounted smartphone. Each subject performed the protocol twice: in the first trial, the smartphone was fixed on the left side of the belt, and in the second, it was placed by the user himself as preferred. Through visual and sound signals, users were informed about the change of activity. The tasks were performed in laboratory conditions, but volunteers were asked to perform the sequence of activities freely for a more naturalistic dataset. In the laboratory, as users were performing each activity, a researcher was annotating them in a mobile application. The result of this annotation was a dataset for each instance (activity performed by each user) with start time, end time, activity, comments (interesting for further works) and user identification. Thanks to this information, data collected on the user smartphone from IMUs could be split and labeled.

Finally, to carry out a comparative analysis of the accuracy and performance of the discrete recognition method proposed in this paper, a new dataset was built. This file contains 6874 overlapped time windows with 170 variables associated with each one and eight activities supported. These activities are standing, walking, running, jumping, cycling, driving, upstairs and downstairs. A group of 10 users with Samsung Galaxy S3. Samsung Galaxy S4 and LG Nexus 5 smartphones participated in the experiment. Table 1 contains information about the users' smartphone distribution along the experiment. Each row shows the phone model used by the user and the number of time windows obtained throughout the whole experiment.

However, far from being a static system, the kind of activities recognized depends on the user. In this line, thanks to the proposed method for new activity detection, introduced later, the system can determine when the users are carrying out activities that had not been learned before. As will be seen later, this is based on the analysis of pattern recognition and identification of low-probability instances.

## 4. Experimental Section

Working in the domain of discrete variables to perform learning and recognition of activities constitutes the innovative contribution offered by this work. Learning algorithms based on continuous variables, which traditionally have been used for this purpose over the years, lack a high complexity. A main aim in this paper is to use an approach based on discrete variables, which reduces this complexity, as will be shown. Therefore, prior to self-recognition and learning, it is necessary to carry out a process of discretization, which is performed through the application of the Ameva algorithm [41].

This algorithm has a number of advantages, chief among them being the small number of intervals generated, which facilitates and reduces the computational cost of the recognition process. It is worth noting that the Ameva algorithm has always been used as a discretization process [42–44]. In this paper, a new method that allows using the algorithm Ameva as a classification method has been developed.

**Figure 4.**
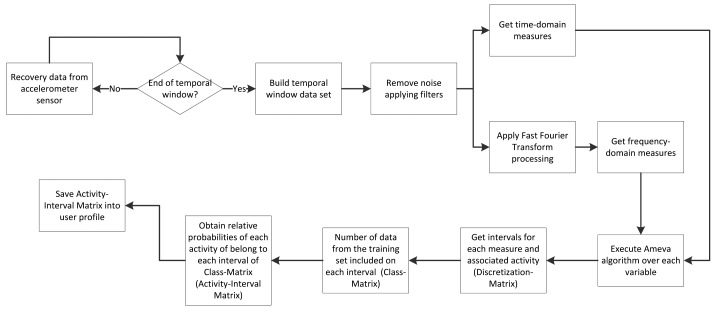
Methodology pipeline for the activity recognition training subsystem.

Figure 4 shows the methodology for the system training. Along this section, each building block is presented, more specifically, the data processing and classification methods. Both make use of discrete techniques for classification, unlike other related works, which usually employ continuous methods [45,46].

### 4.1. Ameva Algorithm

Working in the domain of discrete variables to perform the learning and recognition of activities is a new approach offered by this work. This decision was largely due to the high computational cost required for learning algorithms based on continuous variables that have been used for this purpose over the years.

In [43], a labeling process, like a discretization process, is used to obtain a similarity index, so it can be said that a transformation of the continuous domain to the discrete domain of the values of the variables is beneficial in certain aspects. However, before self-recognition or learning, it is necessary to carry out a process of Ameva discretization from its algorithm [41]. The most notable of these algorithms is the small number of intervals generated, which facilitates and reduces the computational cost of the recognition process.

Let us introduce these algorithms. Let *X* = {*x*_1_,* x*_2_,…,*x_n_*} be a dataset of an attribute *χ* of mixed-mode data, such that each example *x_i_* belongs to only one of the ℓ classes of class variables denoted by 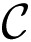 = {*C*_1_, *C*_2_,…, *C*_ℓ_}, ℓ ≥ 2. Table 2 shows a toy dataset with 6 statistics, 10 samples and 3 classes.

A continuous attribute discretization is a function 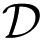 : *χ* → 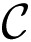, which assigns a class *C_i_* ∈ 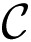 to each value *x* ∈ *χ* in the domain of property that is being discretized. Let us consider a discretization 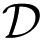 that discretizes *χ* into *k* discrete intervals: ℒ(*k*; *χ*; 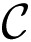) = {*L*_1_, *L*_2_,…,*L_k_*}, where *L*_1_ is the interval [*d*_0_, *d*_1_] and *L_j_* is the interval (*d_j_*_−1_, *d_j_*], *j* = 2,3,… ,*k*. Thus, a discretization variable is defined as ℒ(*k*) = ℒ(*k*; *χ*; 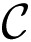), which verifies that, for all *x_i_* ∈ *X*, a unique *L_j_* exists, such that *x_i_* ∈ *L_j_* for *i* = 1, 2,…, *n* and *j* = 1, 2,…, *k*.

The main aim of the Ameva method [41] is to maximize the dependency relationship between the class labels 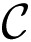 and the continuous-values attribute ℒ(*k*) and, at the same time, to minimize the number of discrete intervals *k*. For this, the following statistic is used:

$$\text{Ameva}\left(k\right)=\frac{\chi^{2}\left(k\right)}{k\left(\ell -1\right)}$$

where:

$$\chi^{2}\left(k\right)=N\left(-1+\sum_{i=1}^{\ell }\sum_{j=1}^{k}\frac{n_{ij}^{2}}{n._{i}n_{j}.}\right)$$

and *n_ij_* denotes the total number of continuous values belonging to the *C_i_* class that are inside the interval *L_j_*, *n_i_*. is the total number of instances belonging to the class *C_i_* and *n._j_* is the total number of instances that belong to the interval *L_j_*, for *i* = 1, 2,…,ℓ and *j* = 1, 2,…, *k*, fulfilling the following:

$$\begin{array}{ccc} n_{i}.=\overset{k}{\underset{j=1}{\text{∑}}}n_{ij}, & n._{j}=\overset{\ell }{\underset{i=1}{\text{∑}}}n_{ij}, & N=\overset{\ell }{\underset{i=1}{\text{∑}}}\overset{k}{\underset{j=1}{\text{∑}}}n_{ij} \end{array}$$

Table 3 shows this interval grouping process over 12,480 instances and 6 intervals. The number of instances contained in each class for a given interval is shown in each row. Columns contain the number of instances inside each interval for a given class. The last row and the last column contain the sum of instances for each class and each interval, respectively.

### 4.2. Discretization Process

Let 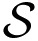 = {*S*_1_, *S*_2_,…, *S_m_*} be a set of *m* statistics. Hence, for each statistic *S_p_* ∈ 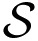, the discretization process is performed, obtaining a matrix of order *k_p_* × 2, where *k_p_* is the number of class intervals and 2 denotes the *inf*(*L_p,i_*) and *sup*(*L_p,i_*) interval limits *i* of the *p* statistic. This three-dimensional matrix containing the set of interval limits for each statistic is called the discretization matrix and is denoted by 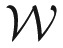 = (*w_pij_*), where *p* = 1, 2, …, *m*, *i* = 1, 2, …, *k_p_* and *j* = 1, 2.

Therefore, the discretization matrix determines the interval at which each datum belongs to the different statistical associated values, carrying out a simple and fast discretization process. Table 4 shows an example of this process.

Appendix A shows the distribution for the first 21 statistics. Each distribution contains the intervals generated on the horizontal axis and the number of associated samples. In this figure, it can be noted that samples are not equally distributed, and the number of intervals is not the same for all statistics. These elements depend on the number of samples for each activity in the dataset and the value distribution.

#### 4.2.1. Class Integration

The aim in the next step of the algorithm is to provide a probability associated with the statistical data for each of the activities based on previously generated intervals. For this purpose, the elements of the training set *x* ∈ *χ* are processed to associate the label of the concrete activity in the training set. In addition, the value of each statistic is calculated based on the time window.

To carry out the previous process, a class matrix, 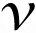, is defined as a three-dimensional matrix that contains the number of data from the training set associated with an ℒ interval in a 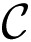 activity for each statistic 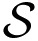 of the system. This matrix is defined as follows: 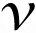 = (*υ_pij_*), where *υ_pij_* = |{*x* ∈ *χ* | *inf*(*L_p,i_*) < *x* ≤ *sup*(*L_p,i_*)}|, and 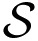 = *S_p_*, 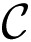 = *C_j_*, *p* = 1, 2, …, *m*, *i* = 1, 2, …, *k_p_* and *j* = 1, 2,…, ℓ.

Thus, each position in 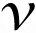 is uniquely associated with a position in 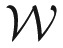 determined by its associated interval. Table 5 shows the contents of a real class matrix obtained during a learning process from a standard deviation statistic (*S_p_*). In this table, the six intervals previously calculated by the Ameva algorithm and stored in 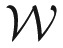 can be observed.

At this point, it is not only possible to determine the discretization interval, but the class matrix also helps to obtain the probability associated with the discretization process performed with the Ameva algorithm.

#### 4.2.2. Activity-Interval Matrix

Now, a matrix of relative probabilities is obtained. This three-dimensional matrix, called the activity-interval matrix and denoted by 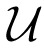, determines the likelihood that a given value *x* associated with an *S* statistic corresponds to 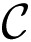 activity in a ℒ interval. This ratio is based on obtaining the goodness of the Ameva discretization, and the aim is to determine the most probable activity from the data and the intervals generated for the training set.

Each value of 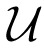 is defined as follows:

$$u_{\text{pij}}=\frac{\upsilon_{\text{pij}}}{\upsilon_{p\cdot j}}\frac{1}{\ell -1}\sum_{q=1,q\neq j}^{\ell }\left(1-\frac{\upsilon_{\text{piq}}}{\upsilon_{p\cdot q}}\right)$$

where *υ_p·j_* is the total number of time windows of the training process labeled with the *j* activity for the *p* statistic, and *p* = 1, 2, …, *m*, *i* = 1, 2, …, *k_p_* and *j* = 1, 2, …, ℓ

Given these values, 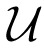 for the *p* statistic is defined as:

$$u_{p}=\left(\begin{array}{ccccc} u_{p00} & \cdots & u_{p0j} & \cdots & u_{p0\ell }\\ \vdots & \ddots & \vdots & \ddots & \vdots \\ u_{pi0} & \cdots & u_{\text{pij}} & \cdots & u_{pi\ell }\\ \vdots & \ddots & \vdots & \ddots & \vdots \\ u_{pk_{p}0} & \cdots & u_{pk_{p}j} & \cdots & u_{pk_{p}\ell } \end{array}\right)$$

Then, the following condition must be considered in order for the above definition to be complete and without errors in the training: $\upsilon_{p\cdot q}=0\to \frac{\upsilon_{\text{piq}}}{\upsilon_{p\cdot q}}=0$.

As can be seen in the definition of 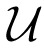, the probability that a datum *x* is associated with the interval *L_i_* corresponding to the activity *C_j_* depends not only on the data, but on all of the elements associated with the interval *L_i_* for the other activities.

Thus, each *u_pij_* matrix position can be considered as the probability that a given *x* belongs to *C_j_* activity, that it is included in the *L_i_* interval of the *S_p_* statistic.

Similarly, the elements of 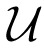 have the following properties:

- *u_pij_* = 0 ⇔ *υ_pij_* = 0 ∨ *υ_piq_* = *υ_p·q_, q* ≠ *j*
- *u_pij_* = 1 ⇔ *υ_pij_* = *υ_p·j_* = *υ_pi_*.

Table 6 shows a set of different values obtained for each of the positions of the activity-interval matrix 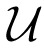. These results were obtained from the training set from the class matrix described in Table .

### 4.3. Classification Process

This section presents the process of classification from the data of the time windows analysis. This process is divided into two main parts. First, the way to perform the recognition of physical activity is described. Later, the task to determine the frequency of a particular activity is exposed.

#### 4.3.1. Classifying Data

For the classification process, the more likely activity is decided by a majority voting system from the activity-interval matrix and a set of data *x* ∈ *X* for the 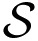 set.

Therefore, it consists of finding an activity *C_i_* ∈ 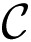 that maximizes the likelihood. The above criterion is collected in the following expression, denoted by *mpa, mpa*(*x*) = *C_k_*, where, $k=\arg\underset{j}{\max}\left(\overset{m}{\underset{p=1}{\text{∑}}}u_{\text{pij}};\text{inf}\left(L_{p,i}\right)<x\leq L_{p,i}\right)$. The expression shows that the weight contributed by each statistic to the likely calculation function is the same. This can be done under the assumption that all statistics provide the same information to the system, and there is no correlation between them. Thus, the most likely activity represents the activity whose data, obtained through the processing time window, are more suited to the value set from the activity-interval matrix.

**Figure 5.**
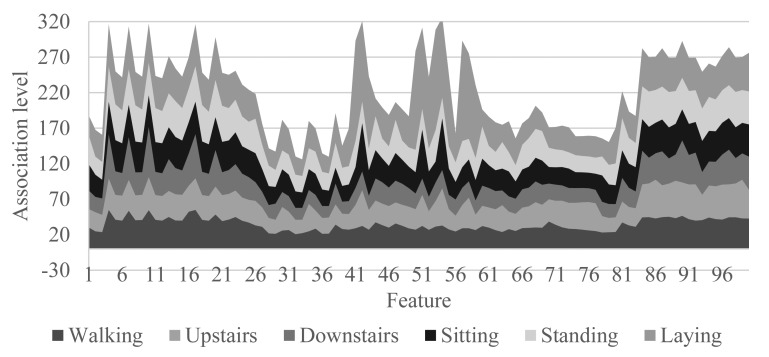
Association level of each activity for the 100 first features.

In this way, the proposed algorithm not only determines the *mpa*, but its associated probability. Figure 5 shows the total interval probability based on a training set and 100 features (Downstairs and upstairs activities make reference to the dynamic activities of ascending and descending stairs). The peaks presented in this chart correspond to the features giving more information to the system. From this likelihood, certain activities that do not adapt well to sets of generic classification can be identified. It is an indication that the user is carrying out new activities for which the system has not been trained previously.

**Figure 6.**
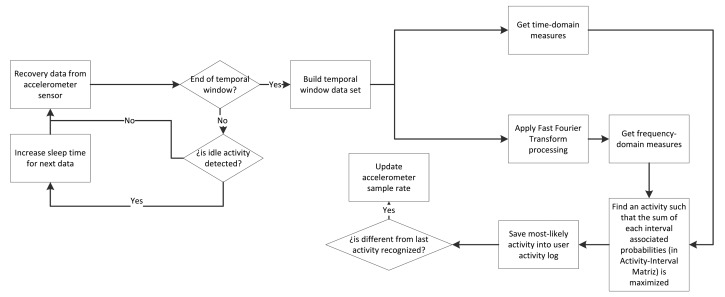
Flow diagram for the recognition process.

Figure 6 shows the process flow described in this section for process recognition from the activity-interval matrix calculated in the previous section.

#### 4.3.2. Activity Cycle Cadence Approach

The cadence at which any activity is performed is really important in order to get the calories burned and the intensity of the recognized activity. For this reason, although this factor does not make contributions to the field of activity recognition systems, it is crucial to get a system with added value applicable in a real life context. To determine the frequency for each activity cycle, the maximum module of the time window frequencies must first be calculated. Subsequently, the frequency associated with the maximum module (*τ*) represents the number of cycles per second for the activity related to the time window processed. From this and by using a simple expression, from the frequency value associated with the activity recognized in the current time window, the frequency of the activities per minute (*z*(*X*)) can be obtained as follows:

z(X)=τ⋅60sec

### 4.4. Dynamic Sample Rate and Duty Cycle

It is crucial to consider energy consumption and the processing cost of the system when it is working on a mobile device. The broadened problem of activity recognition systems based on IMU sensors is the high power consumption caused by keeping the smartphone awake in order to perform all tasks needed, such as sensor sampling and activity classification. The literature has explored three ways of solving this drawback: selecting the system features depending on the computational cost for their calculation [47,48], reducing the sampling rate below the threshold of 50 Hz [49] and implementing a dynamic sampling rate method in the proposed solutions [35,50].

Dynamic sampling rate approaches, the most extensive over the last few years, is based on the demonstration that different activities exhibit differing levels of classification accuracy, depending on the on-body placement of the accelerometers [51]. Through this comparison, the Dynamic Ameva Classification System has been applied, implementing a dynamic sampling rate method for energy reduction. The frequency varies from 32 Hz for sitting, standing and lying to 50 Hz for walking, upstairs and downstairs. It must be taken into account that these frequencies were obtained experimentally by studying the pattern of the different activities from the point of view of the accelerometery. This study was conducted covering over 600,000 different time windows of activities obtained from 10 different users.

To respect the heterogeneity of the target users of the proposed recognition system, users with different profiles were selected, from those 21 years of age with an athletic profile, to seniors 82 years of age with a sedentary profile. Thanks to this information, it is possible to determine a suitable accelerometer frequency spectrum according to each activity. It should be noted that this frequency will have not only a power impact due to the decrease of data obtained from the accelerometer, but will also cause a reduction of the execution time of the algorithm, due to the smaller size of the time windows.

Algorithm 1 calculates the sampling rate corresponding to a recognized activity at a given time. For the calculation, the current recognized activity and previous detected activity are taken into account. If the recognition process enters into a stable phase, *i.e.*, there is continuity in the last set of recognized activities, the algorithm proceeds to update the sample rate. This update is calculated from the sampling rate associated with the activity detected, which is obtained from the map *SampleRateList*. As mentioned before, the specific values for the list *SampleRateList* are calculated experimentally for each of the activities recognized. Later, if stabilization occurs for a prolonged period, our proposal proceeds to the regular updating of the sample rate based on the base log_2_ of the number of memories used.


**Algorithm 1** Dynamic sampling rate algorithm.
 *SampleRateList* ← InitSampleRate(*Frequencies*) *MaxMemoryList* ← InitMemoryList(*Memories*) *AmevaIsRunning* ← true *Count* ← 0 *WinSize* ← 5 *WinSamples* ← 50 * *WinSize*; *A_previous_* ← GetAmevaActivity(*Statistics*) **while**
*AmevaIsRunning*
**do**  *A_last_* ← GetAmevaActivity(*Statistics*)  **if**
*A_last_* == *A_previous_*
**then**   *Count* ← *ActivityMemory* + 1  **else**   *Count* ← 0  **end if**  **if** IsCriticalActivity(*A_last_*) **and**  *Count* > GetMaxMemory(*A_last_*) **then**   *NewFrequency* ← *SampleRateList*(*A_last_*)   *WinSamples* ← *NewFrequency* * *WinSize*  **end if**  **if**
*Count*%30 == *MaxMemoryList*(*A_last_*) **then**   *WinSize* ← *WinSize* + log_2_(*Count*%30)   *WinSamples* ← *NewFrequency* * *WinSize*  **end if**  *A_previous_* ← *A_last_* **end while**


The maximum memory limit is defined as the specific period for which an activity is considered stable. This maximum memory is fixed experimentally and depends on the specific activity. In [52] can be seen a related work, in which the sample rate is calculated from the estimated power required for each sample rate. The main problem of this work is that it does not take into account the specific activity to update the sample rate. Thus, errors may occur due to the low sample rate reached in the case of activity stabilization for a long period. Such excessively low sampling frequencies cause a decrease in the accuracy of the system, as can be seen in [52]. In [53], another system of dynamic frequency is proposed. In this case, the sample rate is only addressed from the activity being performed. This means that at the same time that an activity is recognized, the algorithm immediately proceeds to update the sample rate.

This causes two problems. On the one hand, it could be a case of sporadic activities (e.g., a fall) directly affecting the frequency of sampling and having a negative impact on the next set of activities recognized. On the other hand, by failing to update the sample rate periodically, the ability to minimize power consumption when a long-term stabilization occurs (e.g., when the user goes to sleep) is reduced.

**Figure 7.**
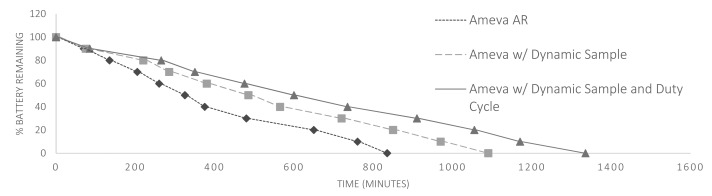
Comparison between simple Ameva method, Ameva with dynamic sample optimization and Ameva with the dynamic sample and duty cycle.

In the proposed algorithm, the size of the time windows is increased, thus decreasing the number of times for carrying out the classification process and, therefore, the consumption caused by the main CPU to perform this action. It should be noted that increasing the size of the temporal window has a direct impact on power consumption, especially in those devices having a coprocessor for context purposes [54]. This CPU, separate from the main CPU, allows autonomous acquisition of contextual data, usually from the accelerometer or gyroscope, without activating the main processor. This produces a decrease in power consumption while these data are collected in the form of an asynchronous batch operation. Thus, by increasing the size of the time window, maximizing the use of the contextual coprocessor and delaying the use of the main CPU for the implementation of the classification algorithm, an increase in the lifetime of the battery is achieved while the recognition system is being used. Keep in mind that this improvement in the use of the coprocessor has not been applied in the comparison with other methods, because it is understood that it may be applied to other comparable jobs with no impact on accuracy. However, it has been key for the lifetime data in the comparison shown in Figure 7, where the three versions of the proposed activity recognition system have been subjected to a cross-comparison.

Furthermore, a second step towards energy savings is introduced by applying idle activity detection. It is well known that for some time, users do not wear their smartphones, so activity recognition is not available. However, during this time, the system is running and, thus, consuming power. Thanks to the idle activity recognition, the system can detect this situation and, consequently, reduce the windows processed per unit time. This decision saves energy and reduces the system overload. For this purpose, when no acceleration is detected on the smartphone, idle mode is enabled.

Algorithm 2 presents this process.


**Algorithm 2** Duty cycle algorithm.
 *DutyCyclePeriods* ← 3,600,000, 1,800,000, 600,000, 300,000, 60,000, 10,000, 3000 *AccDataCollection* ← ∅ *Threshold* ← 0.3 *A_last_* ← Ø *AmevaIsRunning* ← true *WindowsSleep* ← 0 *MinWindowsSleep* ← 5 *DutyIndex* ← 0 *WinSamples* ← WindowLength(*WindowSize*); **while**
*AmevaIsRunning*
**do**  **while**
*size*(*AccDataCollection*) < *WinSamples*
**do**   *AccDataCollection* ← GetLastAccData()  **end while**  *WindowV ar* ← *V ar*(*AccDataCollection*)  **if**
*WindowVar* < *Threshold*
**then**   *A_last_* ← *No_D_evice*   *WindowsSleep* ← *WindowsSleep* + 1   **if**
*WindowsSleep* == *MinWindowsSleep*
**then**    **if**
*DutyIndex* < *size*(*DutyCyclePeriods*) **then**     *DutyIndex* ← *DutyIndex* + 1    **end if**
*Sleep*(*DutyCyclePeriods*[*DutyIndex*])   **end if**  **else**   *DutyIndex* ← 0  **end if** **end while**


First, a number of periods are defined in milliseconds, which will be the values that lead to sleep mode for the activity recognition system. Once the time window is obtained and the variance over this window is calculated, it is determined whether the variance is less than *Threshold*. This *Threshold* was set to 0.3 during the experiments carried out. If the variance exceeds this threshold, this indicates that there has been no significant movement on the device. If so, the *WindowsSleep* varis increased, responsible for counting the number of windows without activity. If *WindowsSleep* is equal to the number of windows needed to decide that the device is in a period of inactivity, given by *MinW indowsSleep*, the algorithm proceeds to the activation or update of the duty cycle. This update consists of a gradual increase, depending on the values of *DutyCyclePeriods*, of the idle time, while it is determined that there is no movement in the successive time windows.

The impact of such optimization in the battery lifetime can be seen in Figure 7. By using the simple Ameva detection, the battery life time reaches 14 h. This is because the system is always on and the accelerometers are working at 50 Hz. Applying the first optimization criteria, the dynamic sampling rate, a battery life of 18 h is obtained, 3 more than previously, because some activities reduce the sampling rate and, therefore, the battery consumption. The last optimization applied to Ameva classification is the idle activity detection. Because most users expend much time at home or working with the device on the table, idle activity detection tunes up the system, avoiding unnecessary energy drain, and the battery lifetime is up to 26 h.

## 5. Results and Discussion

**Figure 8.**
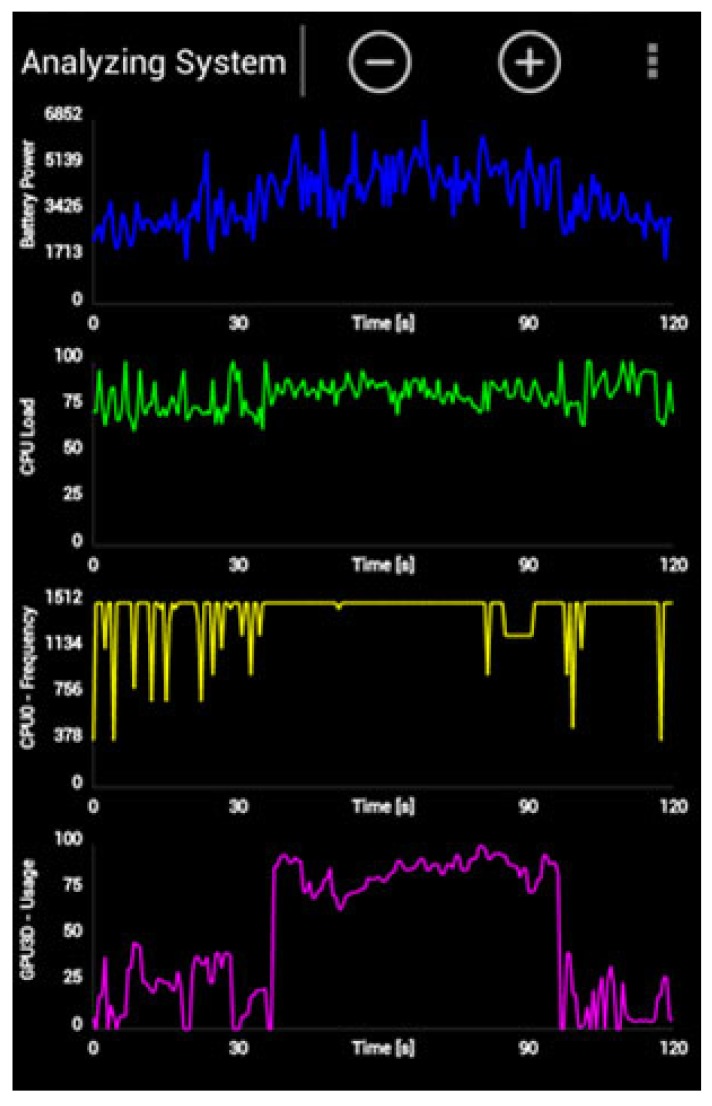
Trepn profiling tool screenshot.

Once the basis of the activity recognition algorithm has been laid out, an analysis of the new proposal can be performed. To this end, the new development is compared with widely-used recognition systems based on neural network, decision tree, SVM and the naive Bayes classifier. These systems have been trained using MATLAB R2014b and implemented on the devices through the Android Studio and SDK tools provided by Google. In order to obtain energy consumption results, as will be explained further below, the monitoring software, Trepn, has been used. Trepn Profiler is an on-target power and performance profiling application for mobile devices distributed by Qualcomm for monitoring snapdragon processors. This diagnostic tool allows profiling of the performance and power consumption of Android applications running under this family of processors. Figure 8 shows a screenshot of this tool. As can be observed in the upper side, battery power is being monitored.

In this case, both learning and recognition are performed by continuous methods. The test process is conducted on Google Nexus S, Samsung Galaxy S3, LG Nexus 5 and Google Nexus One devices for a group of 10 users. Notably, the activity habits of these users are quite different, as two of them are under 25 years old, five users are between 25 and 40 years old and three are over 40. To determine the energy impact of the developed system over the used smartphones, each user was monitored for 15 days. The application ran continuously in the users' smartphones during these 15 days. A total of 3640 h were monitored for all users through the Android application developed.

Furthermore, to perform the accuracy evaluation, each user introduced into the same application the activity that was carried out. In this case, getting users to record their daily activity at all times was very complicated, and hence, a total of 1215 h of registered activities was obtained. From these, a study on the precision was completed. More specifically, the evaluation dataset for each activity is composed as follows: no device (332 h), standing (87 h), walking (198 h), lying (371 h), sitting (215 h), upstairs (5 h) and downstairs (7 h).

### 5.1. Performance Analysis

In this section, the results of the performance analysis comparison between the method presented in this paper and different related works are presented. These methods are Ameva, ANN, binary tree, Bayesian, KNN, SVM, Mahalanobis distance and discriminant analysis. This test is conducted on eight different activities to unify the results for all users. Based on data from the UCI HAR, UCI PAMAP2 and the dataset built in this paper, a more in-depth comparison is performed. To this end, various measures for the evaluation of the performance of the information retrieval system are used. All measures used below assume a ground truth (gold standard value) contained in the labeled datasets. This comparison was performed from the implementation of the algorithms referenced previously. In Table 7, the differences between the compared methods can be observed (These results have been obtained using the UCI HAR dataset, the PAMAP2 dataset and a custom dataset built in this work). Most values presented for each measure show that the Ameva method gives better classification than the other algorithms, especially regarding precision. That is, the number of false positives in the Ameva method is lower than in the others methods. Furthermore, the training process for Ameva was faster than the others, except for binary tree, where the time was very similar. Appendix B shows more in-depth performance measures obtained by applying different algorithms compared in the table above. These values have been separated according to the specific activity. Thus, the accuracy variation can be observed more in detail between the different methods depending on the activity undertaken. However, as can be seen in these tables, there is no great difference between the values obtained in terms of activity. Therefore, it can be concluded that the compared learning methods are robust to the activities under monitoring. Based on the results for error and classification analysis above, it can be determined that the Ameva method for activity recognition presents better results than the other methods, which are widely used throughout the literature, especially SVM and Bayesian. Furthermore, not only is the execution time of the Ameva algorithm faster than that given by the others, the risk of overloading the system under the Ameva method is also lower. This is because a majority vote brings a dynamism that makes certain statistical values not critical when performing the classification, as for example with classification trees.

### 5.2. Energy Consumption Results

A comparison with other works in the field of energy consumption is not easy at all. The main problems are the heterogeneity of smartphones on the market with different batteries, consumption, screens and processors and the use of the smartphone for other tasks, such as calling, reading emails or using WhatsApp. A real analysis without any restriction to the users was carried out. Users utilize theirs smartphones normally. Hence, battery consumption depends on this use. Tests were executed on LG Nexus 5, Samsung Galaxy S3 and Samsung Galaxy S4 devices. The devices were restored to their original configuration after each test to avoid interference from external application consumption.

**Figure 9.**
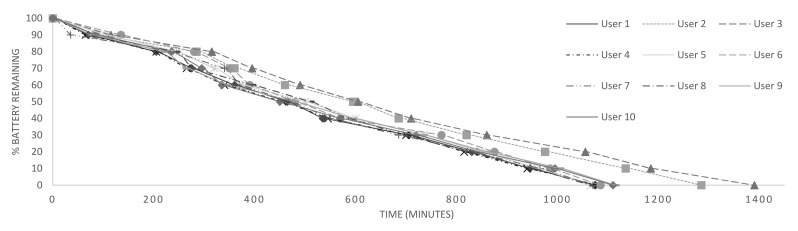
Analysis of battery lifetime through different user habits.

Figure 9 shows the battery lifetime for 10 users by applying the Ameva method with the optimizations described above. As can be seen, battery life depends largely on the users' habits. Whereas, for User 3, the usage time is up to 23 h, for User 4, it is close to 18 h. It must be noted that all users are related to computer science environments, and the use of their devices is quite high.

The current work is now compared with KNN, binary decision tree (C4.5), SVM, neural networks and the naive Bayes classifier. In all cases, the process for obtaining the needed data and calculating the features is the same. This allows comparison of just the computational cost of the classification algorithms.

Table 8 shows the battery lifetime (in minutes) for each classifier, with four tests conducted for each one. As can be observed, the Ameva classifier extends the battery time by three hours compared with the next most efficient method, the binary decision tree (C4.5). In this section, learning times are not taken into account; only the recognition process was evaluated.

**Figure 10.**
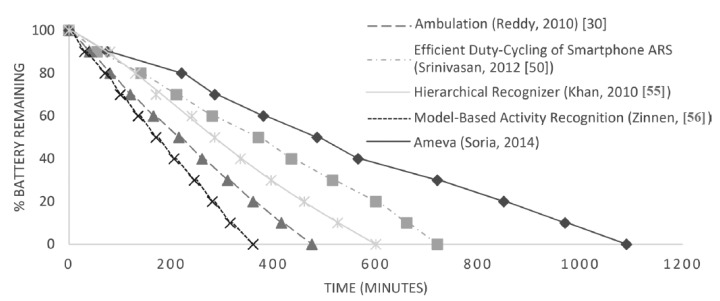
Battery lifetime analysis over other methods.

Finally, this work is compared with other AR systems from the literature [30,50,55,56] implemented and run on the same user's smartphone. Figure 10 shows the results.

**Figure 11.**
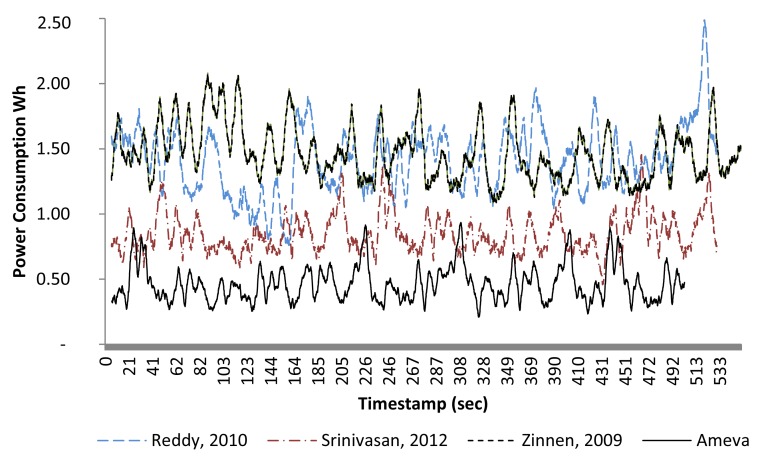
Power consumption analysis.

It can be observed in Figure 10 that the Ameva system increases the battery lifetime by up to 4.5 h. As mentioned before, all methods were executed in the same smartphone used by the same user, each for five days. Later, other works will be compared in terms of accuracy, but for now, we can see that the proposed system significantly reduces the energy consumed. Figure 11 shows a power consumption comparison based on W/h (The watt-hour (W/h) is a unit of energy equivalent to one watt of power expended for one hour) for each compared method. To reduce deviations from the power consumption given by Trepn software, the device was previously calibrated using a spectrum analyzer, which measured the real consumption. Thereby, the correctness of the Trepn Qualcomm software readings was checked. Moreover, because of the impact that CPU consumption obviously has on processing cost, it was decided to keep a wake lock on the device. This ensured that the processor would not enter into a low-power mode during the performance comparison. Thus, the baseline for comparison is one in which the device is not used, but the processors remain awake. It should be noted that this wake lock was deactivated for the battery life tests, in order to perform testing of each algorithm behavior in a real application scenario. As can be seen in Figure 11, the energy consumption of the Ameva algorithm is significantly less than the other alternatives. Specifically, it can be seen that the consumption is about 50% that of the most efficient alternative among those compared. This is mainly due to two reasons: first, the possibility of carrying out a selection of variables at run time, depending on the coefficient Ameva for each statistic; and second, the reduced computational cost of the proposed classification method.

**Figure 12.**
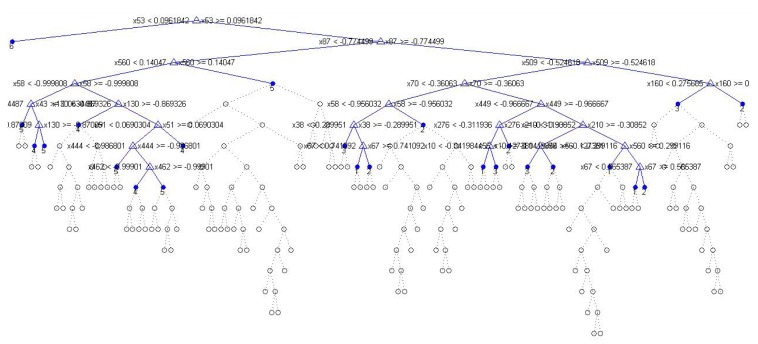
C4.5 bounded decision tree.

To clarify the differences in the power consumption characteristics of different classifiers, these are compared with the proposed algorithm. The C4.5 algorithm will specifically be discussed, which was *a priori* more efficient from the point of view of complexity and which has been used by many studies in the literature. Regarding accuracy, there is no big difference between Ameva and the algorithm based on C4.5, as was shown previously. However, there is a difference from the point of view of energy consumption. Specifically, Ameva is about 50% below the average consumption of the algorithm C4.5. This is mainly due to the capability of automatic selection that is made based on the characteristics of the Ameva coefficient. Those intervals generated by Ameva whose coefficient is less than the threshold (at this moment, the threshold is defined in a static way) are removed, and the associated statistics are not taken into account when computing the result. This makes the used statistic, on average, 40% of the pre-established attributes of each temporal window. This process, applied to the UCIHAR dataset [39], makes the total statistics considered go from 561 to 63, while C4.5 should consider 117 attributes for classification. The tree generated by the C4.5 algorithm can be seen in Figure 12. Consequently, this decrease in the processed attributes makes it unnecessary to process the data on the time window in order to get them and, as a result, reduces the complexity of the process of collecting statistics regarding the C4.5 algorithm.

In conclusion, the power consumption aim of this work has been accomplished. It is noted in [57] that users recharge their smartphones once a day, mostly happening at 8 pm, when users are at home. Because our system allows the user to maintain the device battery for more than 18 hours in all cases, they can retain their recharging habits.

### 5.3. Data Traffic Reduction

Regarding the flow of information between device and server, it has been determined that it is much lower in the case of the Ameva method. This server is responsible for safeguarding the training data and recognized activities. The received data flow necessary for the maintenance of such data is 4.7 Kbytes for the system based on neural networks and 500 bytes in the case of Ameva. That is, the data flow between device and server is reduced by more than 70%. This reduction prevents additional costs arising from excessive use of the data network, because, with the Ameva method, it is only necessary to send the bounds of the intervals. However, using neural networks to train the system, every network parameter and weight must be sent, thereby resulting in a much greater size. The same occurs for all continuous learning methods.

### 5.4. Comparing with Other Works

Once the proposal has been analyzed and optimization has been applied to the original system, a comparison with other proposals for activity monitoring is made in this section. The comparison is based on the following attributes: number of activities, average accuracy, number of sensors, execution environment (the device on which to carry out the recognition), average processing time and battery lifetime. Based on previous data, an analysis of the latest work in the activity recognition field was developed. From all related works, four studies were chosen to carry out the analysis. All four studies are recent and present a large number of citations.

Table 9 shows the results of the comparison. An analysis of the table shows that the number of activities recognized by our proposal is higher than those in the other proposals, except for the Kerem Altun study. However, the Kerem Altun proposal uses five specific sensors, whereas our proposal uses only one sensor embedded in the user's own device.

The Chang Goo and Tanzeem Choudour proposals use just one sensor, but the accuracy is lower than that presented in this paper. Furthermore, the possibility of integrating the whole recognition system inside a mobile phone renders the device more convenient for users.

Another aspect to consider in the comparison is the efficiency of the methods at performing the whole process. In this sense, it is necessary to differentiate between two types of proposals: smartphone-embedded methods and server methods. In the former, the process is executed entirely in the mobile phone, whereas in the latter, a computer is required to execute the solution and to process the data. For this reason, in the Kerem Altun and Chang Goo proposals, the battery life is longer than that in our proposal, which collects and processes the data in the device itself.

Andreas Zinnen marked a new point of view of activity recognition, called model-oriented methods. In that work, some accelerometers are placed on the user's arm, the aim being to recognize the movements made by the body like a three-dimensional model of the user. This technique is often used in animation, but the main drawback is the number of sensors needed. Furthermore, one of the aims of this work is to develop the entire system in the user's smartphone, without external sensors. However, as can be seen in [56], the number of activities recognized is quite high.

Finally, Jia's work introduces other external sensors, such as the ECG meter, which improves the accuracy of the whole system. However, this kind of system has a drawback: the power consumption caused by the Bluetooth connection between external sensors and the smartphone.

## 6. Conclusions and Future Work

This work presents a highly accurate recognition system, based on discrete variables, that uses the Ameva discretization algorithm and a new Ameva-based classification system. It has therefore been possible to achieve an average accuracy of 98% for the recognition of eight types of activities. Furthermore, working with discrete variables significantly reduces the computational cost associated with data processing during the recognition process. By using this process to increase recognition frequency, it has been possible to obtain a physical activity reading every four seconds and to save this contextual information in the user activity live log.

The main problem detected in the system based on statistical learning is the limitation of the number of activities that can be recognized. Actually, the problem is not provoked by the method itself, but by the accelerometer sensors. The number of system features is limited, thus leading to a strong correlation between these variables. This problem could be solved by including new sensors (NFC, Bluetooth, and so on), which provide more information to the system.

Based on the studies performed and the conclusions reached in the Dynamic Sample Rate and Duty Cycle section, the accuracy of the system, once duty cycle optimization is applied, does not vary depending on the user who performs the test. As was mentioned before, this method has a very slight impact on system accuracy. This is because its auto-reconfiguration makes it possible to increase the sample rate if necessary. However, this strategy brings considerable benefit in terms of the energy savings achieved.

In this way, a system that extends the number of recognized activities is currently being developed. It is based on the data presented in this work combined with the help of GPS and NFC sensors embedded in the device. The system involves the analysis of labels installed in smart items that, in addition to providing information about the item itself, inform the system about the activities supported. Therefore, if a user is sitting near the television remote control, then the new activity recognition would be watching TV. Similarly, if a user is walking and GPS information indicates that the user is in the park, the activity would be walking through the park.

## Figures and Tables

**Table 1.** Distribution of users' smartphones during the experiment.

**User**	**Phone Model**	**Time Windows Collected**
1	Samsung Galaxy S3	740
2	Samsung Galaxy S4	683
3	Samsung Galaxy S3	830
4	LG Nexus 5	716
5	Samsung Galaxy S3	519
6	LG Nexus 5	683
7	Samsung Galaxy S4	478
8	Samsung Galaxy S4	854
9	Samsung Galaxy S3	729
10	LG Nexus 5	642

**Table 2.** Example dataset with 6 statistics, 10 samples and 3 different classes.

**Statistics**						***Class***

**Mean**	**Std Deviation**	**Maximum**	**Minimum**	**Energy**	**Skewness**	
10.1	3.9	5.1	13.3	9.7	1.9	*C*_1_
12.7	8.6	3.1	16.4	16.2	0.1	*C*_2_
8.3	1.5	8.3	9.5	1.8	−2.4	*C*_3_
11.3	4.1	6.3	14.9	11.2	1.1	*C*_1_
8.6	1.2	8.7	9.1	1.2	1.3	*C*_3_
9.8	2.7	6.5	13.2	9.7	1.7	*C*_1_
14.7	9.2	3.6	15.3	17.1	−0.2	*C*_2_
11.7	8.5	2.9	16.8	14.3	−1.7	*C*_2_
10.6	3.6	5.1	13.8	11.2	0.8	*C*_1_
9.2	0.7	8.9	9.7	0.9	− 1.8	*C*_3_

**Table 3.** Number of values of each *C_i_* class contained in each interval *L_j_*.

**Interval**	**Class**			***n****_i_*.

	***C*_1_**	***C*_2_**	***C*_3_**	
*L*_1_	3213	65	1	3279
*L*_2_	412	156	4	572
*L*_3_	318	891	86	1295
*L*_4_	136	2178	312	2626
*L*_5_	49	710	813	1572
*L*_6_	0	13	3,123	3136

*n._j_*	4128	4013	4339	12,480 (*N*)

**Table 4.** Example of a discretization matrix.

**Interval**	**Limit**	

	***inf* (*L****_p,i_***)**	***sup*(*L****_p,i_***)**
*L_p_*_,1_	−∞	−0.99
*L_p_*_,2_	−0.99	−0.98
*L_p_*_,3_	−0.98	−0.66
*L_p_*_,4_	−0.66	−0.28
*L_p_*_,5_	−0.28	0.02
*L_p_*_,6_	0.02	+∞

**Table 5.** Example of class matrix 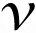 for 6 discretization intervals and 6 activities.

**Interval**	**Activity**					

	**Walking**	**Upstairs**	**Downstairs**	**Sitting**	**Standing**	**Lying**
*L_p_*_,1_	0	0	0	440	524	124
*L_p_*_,2_	0	0	0	367	351	388
*L_p_*_,3_	3	0	0	349	362	734
*L_p_*_,4_	690	375	24	1	0	17
*L_p_*_,5_	394	534	226	0	0	3
*L_p_*_,6_	17	57	637	0	0	0

Total	1104	966	887	1157	1237	1266

**Table 6.** Activity-interval matrix 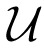 for the standard deviation with 6 discretization intervals and 6 activities.

**Interval**	**Activity**					

	**Walking**	**Upstairs**	**Downstairs**	**Sitting**	**Standing**	**Lying**
*L_p_*_,1_	0.00	0.00	0.00	0.42	0.48	0.10
*L_p_*_,2_	0.00	0.00	0.00	0.35	0.31	0.34
*L_p_*,_3_	0.00	0.00	0.00	0.25	0.24	0.51
*L_p_*_,4_	0.61	0.36	0.02	0.00	0.00	0.01
*L_p_*_,5_	0.30	0.49	0.21	0.00	0.00	0.00
*L_p_*_,6_	0.02	0.07	0.92	0.00	0.00	0.00

**Table 7.** Performance comparison in % by using measures of evaluation.

**Method**	**Accuracy**	**Recall**	**Specificity**	**Precision**	***F*_1_-Measure**
Ameva	99.23	96.93	99.56	96.94	96.93
ANN	98.36	93.50	99.07	93.47	93.48
C4.5	97.51	90.08	98.58	90.04	90.03
Bayesian	92.56	70.96	95.75	70.22	70.41
KNN	97.66	90.74	98.66	90.66	90.69
SVM	90.56	63.09	94.61	62.22	62.41
Mahalanobis distance	93.48	87.41	93.87	91.74	91.67
Discriminant analysis	99.15	96.14	97.75	96.37	96.28

**Table 8.** Battery lifetime in minutes for the execution with different classifiers.

**Algorithm**	**Test**				**Mean**

	**1**	**2**	**3**	**4**	
Ameva	1130	1150	1070	1205	1138
*KNN*	750	730	760	700	731
Binary decision tree (C4.5)	915	925	898	907	905
*SVM*	820	870	780	830	822
Neural Networks	515	597	578	510	548
Naive Bayes	780	750	820	870	814

**Table 9.** Comparison with other activity recognition systems. ND (not determined). * These results are a little confusing. Most smartphones' batteries rarely reach up to 120 h making intensive use of the accelerometer. Furthermore, only two activities (stable or moving) are recognized. ** Making intensive use of Bluetooth.

**Method**	**No. of Activities**	**Average Accuracy (%)**	**No. of Sensors**	**Execution Environment**	**Average Process Time (s)**	**Battery Life (h)**
Our proposal 2014	9	98	1 general	Smartphone	0.3	18 (measured)
Kerem Altun [21] 2010	16	97	5 specific	AMD Athlon 64 X2	0.1	Not applicable
Chang Goo Han [11] 2010	6	92	1 specific	PC	ND	Not applicable
Tanzeem Choudhury [36] 2008	8	84	2 specific	Specific device	ND	10–20 (experimental)
Sasank Reddy [30] 2010	5	93	8 general	Phone	15.0	8.2 (measured)
Hong Lu [49] 2010	5	94	1 general	Smartphone	ND	14 (experimental)
Vijay Srinivasan [50] 2012	6	91	1 general	Smartphone	0.6	12.5 (measured)
Khan [55] 2010	7	97	1 specific	Specific device	2.3	10 (measured)
Yi Wang [58] 2012	2	90	1 general	Phone	ND	150 (experimental) (*)
Jia [59] 2013	7	98	2 specific	Smartphone	1.5	7 (measured) (**)
Andreas Zinnen [56] 2009	21	85	5 general	Smartphone	ND	6.5 (measured) (**)

**Figure A1.**
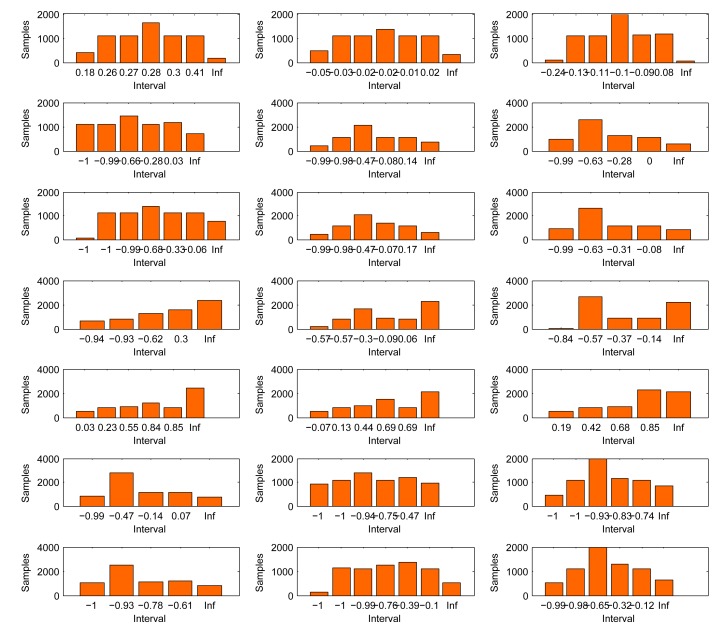
Distribution of instances in each interval for the first 21 statistics.

## Appendix

### Appendix B

**Table B1.** Confusion matrix and performance values for the Ameva-based classification system.

**Test/Real**	**Walk**	**Jump**	**Immobile**	**Run**	**Up**	**Down**	**Cycle**	**Drive**	**Instances**	**TP**	**TN**	**FP**	**FN**	**Accuracy**	**Recall**	**Specificity**	**Precision**	*F*_1_**-Measure**
Walk	7200	10	10	61	80	40	37	89	7527	7200	52,135	327	291	98.97%	96.12%	99.38%	95.66%	95.88%
Jump	10	7304	0	152	0	0	21	8	7495	7304	52,180	191	278	99.22%	96.33%	99.64%	97.45%	96.89%
Immobile	0	0	7256	0	0	0	0	142	7398	7256	52,529	142	26	99.72%	99.64%	99.73%	98.08%	98.86%
Run	16	128	0	7298	0	7	40	8	7497	7298	52,205	199	251	99.25%	96.68%	99.62%	97.35%	97.01%
Up	89	70	0	8	7273	195	2	2	7639	7273	51,918	366	396	98.73%	94.84%	99.30%	95.21%	95.02%
Down	96	0	0	23	316	7252	4	5	7696	7252	52,015	444	242	98.86%	96.77%	99.15%	94.23%	95.48%
Cycle	0	0	0	0	0	0	7302	1	7303	7302	52,536	1	114	99.81%	98.46%	100.00%	99.99%	99.22%
Drive	80	70	16	7	0	0	10	7215	7398	7215	52,300	183	255	99.27%	96.59%	99.65%	97.53%	97.05%

Accumulated	7491	7582	7282	7549	7669	7494	7416	7470	59,953	58,100	417,818	1853	1853	99.23%	96.93%	99.56%	96.94%	96.93%

**Table B2.** Confusion matrix and performance values for the ANN-based classification system.

**Test/Real**	**Walk**	**Jump**	**Immobile**	**Run**	**Up**	**Down**	**Cycle**	**Drive**	**Instances**	**TP**	**TN**	**FP**	**FN**	**Accuracy**	**Recall**	**Specificity**	**Precision**	*F*_1_**-Measure**
Walk	6930	46	41	117	107	79	86	121	7527	6930	51,807	597	619	97.97%	91.80%	98.86%	92.07%	91.93%
Jump	76	7124	16	187	13	8	46	25	7495	7124	51,892	371	566	98.44%	92.64%	99.29%	95.05%	93.,83%
Immobile	47	9	6970	4	63	46	13	246	7398	6970	52,307	428	248	98.87%	96.56%	99.19%	94.21%	95.37%
Run	44	221	3	7060	39	43	59	28	7497	7060	52,016	437	440	98.54%	94.13%	99.17%	94.17%	94.15%
Up	126	89	17	25	7091	256	19	16	7639	7091	51,587	548	727	97.87%	90.70%	98.95%	92.83%	91.75%
Down	103	4	7	27	415	7103	16	21	7696	7103	51,754	593	503	98.17%	93.39%	98.87%	92.29%	92.84%
Cycle	36	32	43	35	75	56	6928	98	7303	6928	52,386	375	264	98.93%	96.33%	99.29%	94.87%	95.59%
Drive	187	165	121	45	15	15	25	6825	7398	6825	52,000	573	555	98.12%	92.48%	98.91%	92.25%	92.37%

Accumulated	7549	7690	7218	7500	7818	7606	7192	7380	59,953	56,031	415,749	3922	3922	98.36%	93.50%	99.07%	93.47%	93.48%

**Table B3.** Confusion matrix and performance values for the C4.5-based classification system.

**Test/Real**	**Walk**	**Jump**	**Immobile**	**Run**	**Up**	**Down**	**Cycle**	**Drive**	**Instances**	**TP**	**TN**	**FP**	**FN**	**Accuracy**	**Recall**	**Specificity**	**Precision**	*F*_1_**-Measure**
Walk	6210	78	98	318	298	96	92	337	7527	6210	51,570	1317	856	96.38%	87.89%	97.51%	82.50%	85.11%
Jump	113	6932	54	245	36	17	67	31	7495	6932	51,645	563	813	97.70%	89.50%	98.92%	92.49%	90.97%
Immobile	59	15	6790	23	83	72	59	297	7398	6790	52,073	608	482	98.18%	93.37%	98.85%	91.78%	92.57%
Run	54	294	23	6896	64	57	71	38	7497	6896	51,649	601	807	97.65%	89.52%	98.85%	91.98%	90.74%
Up	160	113	51	34	6943	298	21	19	7639	6943	51,272	696	1042	97.10%	86.95%	98.66%	90.89%	88.88%
Down	187	18	15	53	427	6917	43	36	7696	6917	51,600	779	657	97.60%	91.33%	98.51%	89.88%	90.60%
Cycle	64	79	54	66	96	74	6716	154	7303	6716	52,241	587	409	98.34%	94.26%	98.89%	91.96%	93.10%
Drive	219	216	187	68	38	43	56	6571	7398	6571	51,643	827	912	97.10%	87.81%	98.42%	88.82%	88.31%

Accumulated	7066	7745	7272	7703	7985	7574	7125	7483	59,953	53,975	413,693	5978	5978	97.51%	90.08%	98.58%	90.04%	90.03%

**Table B4.** Confusion matrix and performance values for the Bayesian-based classification system.

**Test/Real**	**Walk**	**Jump**	**Immobile**	**Run**	**Up**	**Down**	**Cycle**	**Drive**	**Instances**	**TP**	**TN**	**FP**	**FN**	**Accuracy**	**Recall**	**Specificity**	**Precision**	*F*_1_**-measure**
Walk	5067	187	218	573	421	329	198	534	7527	5067	49,701	2460	2725	91.35%	65.03%	95.28%	67.32%	66.15%
Jump	741	5180	113	1023	57	124	216	41	7495	5180	49,239	2315	3219	90.77%	61.67%	95.51%	69.11%	65.18%
Immobile	115	87	5014	288	356	298	243	997	7398	5014	51,473	2384	1082	94.22%	82.25%	95.57%	67.78%	74.31%
Run	78	1520	46	5290	155	96	245	67	7497	5290	50,047	2207	2409	92.30%	68.71%	95.78%	70.56%	69.62%
Up	613	509	79	77	5521	708	56	76	7639	5521	49,478	2118	2836	91.74%	66.06%	95.90%	72.27%	69.03%
Down	470	27	43	76	1309	5641	76	54	7696	5641	50,323	2055	1934	93.35%	74.47%	96.08%	73.30%	73.88%
Cycle	105	127	145	227	451	300	5216	732	7303	5216	51,511	2087	1139	94.62%	82.08%	96.11%	71.42%	76.38%
Drive	603	762	438	145	87	79	105	5179	7398	5179	50,054	2219	2501	92.13%	67.43%	95.75%	70.01%	68.70%

Accumulated	7792	8399	6096	7699	8357	7575	6355	7680	59,953	42,108	401,826	17,845	17,845	92.56%	70.96%	95.75%	70.22%	70.41%

**Table B5.** Confusion matrix and performance values for the SVM-based classification system.

**Test/Real**	**Walk**	**Jump**	**Immobile**	**Run**	**Up**	**Down**	**Cycle**	**Drive**	**Instances**	**TP**	**TN**	**FP**	**FN**	**Accuracy**	**Recall**	**Specificity**	**Precision**	*F*_1_**-Measure**
Walk	4562	210	289	658	513	401	201	693	7527	4562	48,751	2965	3675	88.92%	55.38%	94.27%	60.61%	57.88%
Jump	819	4716	214	1142	64	176	275	89	7495	4716	48,621	2779	3837	88.96%	55.14%	94.59%	62.92%	58.77%
Immobile	156	115	4663	342	398	341	296	1087	7398	4663	51,018	2735	1537	92.87%	75.21%	94.91%	63.03%	68.58%
Run	89	1598	85	4982	174	158	335	76	7497	4982	49,516	2515	2940	90.90%	62.89%	95.17%	66.45%	64.62%
Up	769	654	97	85	4728	1127	82	97	7639	4728	48,875	2911	3439	89.41%	57.89%	94.38%	61.89%	59.83%
Down	636	53	68	84	1614	5092	87	62	7696	5092	49,504	2604	2753	91.06%	64.91%	95.00%	66.16%	65.53%
Cycle	354	297	241	361	580	467	4189	814	7303	4189	51,120	3114	1530	92.25%	73.25%	94.26%	57.36%	64.34%
Drive	852	910	543	268	96	83	254	4392	7398	4392	49,637	3006	2918	90.12%	60.08%	94.29%	59.37%	59.72%

Accumulated	8237	8553	6200	7922	8167	7845	5719	7310	59,953	37,324	397,042	22,629	22,629	90.56%	63.09%	94.61%	62.22%	62.41%

**Table B6.** Confusion matrix and performance values for the KNN-based classification system.

**Test/Real**	**Walk**	**Jump**	**Immobile**	**Run**	**Up**	**Down**	**Cycle**	**Drive**	**Instances**	**TP**	**TN**	**FP**	**FN**	**Accuracy**	**Recall**	**Specificity**	**Precision**	*F*_1_**-Measure**
Walk	6726	65	52	143	137	87	121	196	7527	6726	51,490	801	936	97.10%	87.78%	98.47%	89.36%	88.56%
Jump	161	6879	26	265	36	27	62	39	7495	6879	51,768	616	690	97.82%	90.88%	98.82%	91.78%	91.33%
Immobile	65	26	6719	21	95	64	26	382	7398	6719	52,193	679	362	98.26%	94.89%	98.72%	90.82%	92.81%
Run	86	244	36	6854	65	79	84	49	7497	6854	51,834	643	622	97.89%	91.68%	98.77%	91.42%	91.55%
Up	149	98	32	41	6881	375	36	27	7639	6881	51,317	758	997	97.07%	87.34%	98.54%	90.08%	88.69%
Down	187	21	16	31	504	6870	26	41	7696	6870	51,522	826	735	97.40%	90.34%	98.42%	89.27%	89.80%
Cycle	65	51	58	47	124	78	6748	132	7303	6748	52,254	555	396	98.41%	94.46%	98.95%	92.40%	93.42%
Drive	223	185	142	74	36	25	41	6672	7398	6672	51,689	726	866	97.34%	88.51%	98.61%	90.19%	89.34%

Accumulated	7662	7569	7081	7476	7878	7605	7144	7538	59,953	54,349	414,067	5604	5604	97.66%	90.74%	98.66%	90.66%	90.69%
